# Polyploid cancer cells surviving cisplatin reallocate central carbon sources to fuel antioxidant metabolism for survival

**DOI:** 10.1016/j.molmet.2026.102370

**Published:** 2026-04-18

**Authors:** Melvin Li, Bradley Priem, Luke V. Loftus, Michael J. Betenbaugh, Kenneth J. Pienta, Sarah R. Amend

**Affiliations:** 1 Cancer Ecology Center, The James Brady Urological Institute, Johns Hopkins School of Medicine, Baltimore, MD, 21287, USA; 2 Pharmacology and Molecular Sciences Program, Johns Hopkins School of Medicine, Baltimore, MD, 21287, USA; 3 Department of Chemical and Biomedical Engineering, Johns Hopkins University, Baltimore, MD, 21218, USA

**Keywords:** Chemotherapy resistance, Cancer metabolism, ^13^C-metabolic flux analysis, Genome scale metabolic modeling, Integrated fluxomics

## Abstract

Therapy resistance is the leading cause of cancer-related deaths. Polyploid cancer cells mediate resistance through adaptive cell states transitions that promote survival and tumor recurrence. Here, we investigate metabolic differences between cisplatin-surviving polyploid cells and parental cancer cells using integrated fluxomics. Transcriptomic and proteomic profiling and extracellular flux analyses revealed that surviving cells upregulate glycolysis and gluconeogenesis while reducing oxidative phosphorylation, indicating a shift in central carbon metabolism. Isotope tracing and metabolic modeling demonstrate that surviving cells utilize glucose to fuel the pentose phosphate pathway (PPP) for NADPH generation and metabolize glutamine to provide carbons for the PPP via gluconeogenesis. Integrating our multi-omic datasets into a genome-scale model identified that surviving cells sustain antioxidant metabolism by decreasing fluxes of other NADPH-consuming reactions upon in silico PPP knockout. In addition, pathway-centric transcriptomic analysis revealed that high PPP and antioxidant gene expression correlated with poor survival outcomes in patients across multiple cancer types, demonstrating the clinical prognostic value of PPP and antioxidant metabolism. These findings reveal a systems-level shift in metabolism that maintains antioxidant activity for cell survival, highlighting potential targets and treatment paradigms to overcome therapy resistance.

## Highlight

• Cisplatin-surviving PC3 cells increase glucose uptake and shuttle it into the pentose phosphate pathway (PPP).
• Glutamine supplies carbons for gluconeogenesis, fueling the PPP in cisplatin-surviving PC3 cells.
• The PPP generates 96% of intracellular NADPH in cisplatin-surviving PC3 cells.
• PPP-mediated NADPH sustains antioxidant metabolism in cisplatin-surviving PC3 cells.

## 1 Introduction

Therapy resistance is responsible for 90% of cancer-related deaths and remains a major barrier to cancer cure [1]. Genotoxic chemotherapies, ranging from platinum-based drugs, antimetabolites, and topoisomerase inhibitors, are standard systemic treatments used across all types of solid and blood cancers [2]. While these drugs are initially effective in reducing tumor burden, subpopulations of cancer cells that survive treatment remain, ultimately leading to recurrence and poor prognosis in patients. Classic models of therapy resistance are explained through tumor cell heterogeneity, suggesting that treatment selects for pre-existing clonal populations harboring specific genetic mutations that confer resistance [2]. In recent years, cancer cells have been shown to also adopt non-genetic mechanisms of resistance that are driven by epigenetic and phenotypic plasticity [2].

Our group and others have previously demonstrated an adaptive resistance model in which cancer cells enter a non-proliferative, polyploid cell state in response to genotoxic therapies [[3], [4], [5], [6], [7], [8], [9], [10]]. Cells in this state exhibit increases in cell size and genomic content while maintaining proliferative arrest for weeks after treatment. In addition to emerging in vitro, this cell state has been identified in mouse models and human tumors across a wide range of cancer types [8,[11], [12], [13]]. It can be induced by multiple classes of chemotherapy and targeted therapy, and its presence predicts progression to metastatic disease and poor prognosis [8,10,11,14]. Our previous work has shown that cells surviving in this state exhibit increased mitochondrial fragmentation, enhanced susceptibility to ferroptosis through dysregulated iron homeostasis, and upregulation of lipid droplets in response to oxidative stress – features that collectively suggest the acquisition of metabolic adaptations [[15], [16], [17]].

Cell metabolism is an adaptive process that responds dynamically to nutrient availability, energy demand, and various sources of stress [18,19]. It consists of a complex network linking the epigenome, transcriptome, proteome, metabolome, and fluxome [[20], [21], [22], [23]]. This network is continually shaped by factors such as interactions with the extracellular environment, differential utilization of carbon and nitrogen sources, and rewiring of pathway fluxes [24]. Cancer cells adopt transient metabolic states to allocate resources to grow, proliferate, and resist standard-of-care therapies [25,26]. Depending on the tissue of origin, type of stressors the tumors experience, and disease status, different metabolic pathways can be utilized to fulfill the needs of rapid proliferation and cancer cell survival [27].

In this study, we employ multidisciplinary approaches from cancer cell biology, analytical chemistry, and systems biology to investigate the metabolic alterations in the cancer cells that survive cisplatin treatment. We demonstrate through multi-omic analyses that cisplatin-surviving cells increase glucose and glutamine uptake, but rather than shuttling them into ATP-generating pathways, they allocate those carbon sources to generate antioxidants for cell survival. Characterization of the metabolic transcriptome and proteome in surviving cells revealed increase expression of key enzymes involved in glycolysis and gluconeogenesis while decreasing expression of oxidative phosphorylation (OXPHOS)-related proteins. Through isotope tracing and ^13^C-metabolic flux analysis (^13^C-MFA), we quantitatively mapped metabolic flux differences between surviving cells and parental cells, showing the rewiring of glucose and glutamine to fuel the oxidative pentose phosphate pathway (oxPPP) in surviving cells. We then integrated our proteomic and ^13^C-MFA datasets into a genome scale metabolic model to assess the role of pentose phosphate pathway-derived NADPH on antioxidant metabolism, providing a robust systems-level view on metabolic reprogramming in cancer cells surviving cisplatin. In silico knockout of the oxPPP in surviving cells revealed a possible compensatory mechanism to sustain antioxidant capacity through the reduction of folate metabolism. In addition, we find that high expression of oxPPP and antioxidant-related genes in human tumors predicted worse overall survival outcomes across three cancer types from publicly available datasets. Our findings provide new insights into the metabolic flexibility of cisplatin-surviving cells and highlight potential vulnerabilities to target this transient resistant state.

## 2 Materials and methods

### 2.1 Cell culture and induction of resistant cancer cell state

PC3 parental cells were cultured in RPMI 1640 medium (Thermo Fisher Scientific; Cat# 11875119) with 10% fetal bovine serum (FBS) (Avantar; Cat# 97068-085) and 1% penicillin and streptomycin (P/S) (Gibco^TM^; Cat#15140-122). Cells were incubated at 37 °C and 5% CO_2_. To induce the resistant cancer cell state, cells were treated with the LD_50_ dose of cisplatin (6 μM) for 72 h. Drug-containing media was removed, and cells were left in culture for 10 days, with media changes every 3 days. Cells at 10 days post-treatment removal (10 Days PTR) and parental cells were used for experiments.

### 2.2 Single-cell RNA sequencing analysis

**Figure 1:**
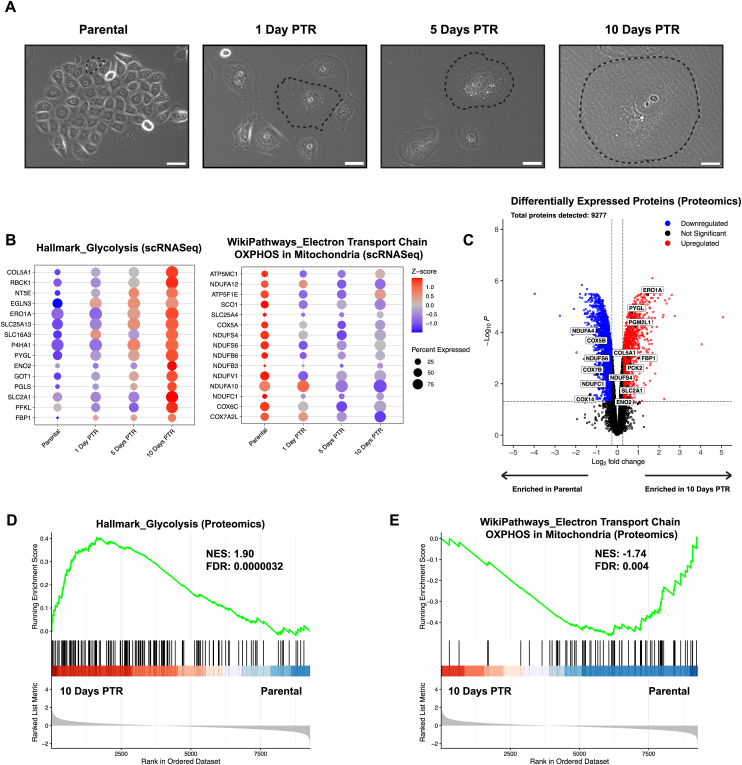
**Cells that survive cisplatin treatment exhibit transcriptomic and proteomic signatures of increased glucose metabolism and decreased oxidative phosphorylation**. (**A**) Phase contrast images of parental PC3 cells and cells 1-, 5-, and 10 Days Post-Treatment Removal (PTR). Black dotted lines indicate the cell border of parental cells and cells 1-, 5-, and 10 Days Post-Treatment Removal (PTR). Scale bar: 100 μm. (**B**) Single-cell RNA expression bubble plots of leading-edge genes from Hallmark Glycolysis and Wikipathways Electron Transport Chain OXPHOS in Mitochondria gene sets. (**C**) Volcano plot of differentially expressed proteins in cells 10 Days PTR vs parental cells. Log_2_ fold change cutoff was 0.263 and adjusted p-value cutoff was 0.05. (**D**) Hallmark Glycolysis proteomic pathway enrichment analysis between cells 10 Days PTR and parental cells. (**E**) WikiPathways Electron Transport Chain OXPHOS in Mitochondria proteomic pathway enrichment analysis between cells 10 Days PTR and parental cells. n = 3 biological replicates for parental cells and cells 1-, 5-, and 10 Days PTR (**B**). n = 4 biological replicates for parental cells and cells 10 Days PTR (**C-E**). Adjusted p-values were generated in R using Benjamini-Hochberg correction (**C**). False discovery rates (FDR) and normalized enrichment scores (NES) were generated for pathway enrichment analyses in R. FDR cutoff was set to 0.05 (**D-E**).

Transcriptomic data of parental cells and cells 1-,5-, and 10 Days PTR was previously deposited by our group into the Gene Expression Omnibus (GEO) database (GSE297299). Data was imported into Partek Flow software (Illumina) for normalization, z-score expression calculations, and downstream analysis. Results were exported as CSV files and plotted using R with the ggplot2 package [28]. Data outputs from these analyses are in Source Data Figure 1 and Source Data Figure 6.

### 2.3 Protein lysate harvest for bulk proteomics

Parental cells and cells 10 Days PTR were lifted from T150 flasks with TrypLE^TM^ Express (Gibco^TM^; Ref# 12604-013) and lysed using RIPA buffer (Sigma–Aldrich; Cat#: R0278-500 ML) with 1X Halt Protease & Phosphatase cocktail and 0.005 M EDTA (Thermo Fisher Scientific; Cat#: 78440). Lysates were sonicated in a Branson 3510 Ultrasonic Cleaner for 10 min and then were subsequently spun in a 4 °C centrifuge at 21,000 rcf. Supernatant was collected and stored at −20 °C. The Pierce^TM^ BCA Protein Assay (Thermo Fisher Scientific; Cat#: 23227) was used to quantify protein concentration, and 10 μg of protein was used for bulk proteomics.

### 2.4 Sample preparation for bulk proteomics

Protein extracts were buffer exchanged using SP3 paramagnetic beads (GE Healthcare) [29]. Briefly, 10 μg of protein was resolubilized in 10 mM TEAB and disulfide bonds reduced with dithiothreitol (5 mM final concentration) for 1 h at 60 °C. Samples were cooled to room temperature and pH adjusted to ∼8.0, followed by alkylation with iodoacetamide (10 mM final concentration) in the dark at room temperature for 15 min. Next, SP3 beads were added to the samples at a ratio of 10:1 bead:protein and 100% ethanol was added to achieve a final ethanol concentration of 50% v/v. Samples were incubated at room temperature with shaking for 5 min. Following protein binding, beads were washed with 180 μL 80% ethanol three times. Proteins were digested on-bead with trypsin (Pierce) at 37 °C overnight at a ratio of 10:1 protein:enzyme. Resulting peptides were separated from the beads using a magnetic tube holder. Supernatants containing peptides were removed from the beads and dried using vacuum centrifugation.

### 2.5 Isobaric tandem mass tag (TMT) labeling

Peptides were labeled with TMTpro reagents (Thermo Fisher Scientific) according to the manufacturer's instructions. After quenching of the labeling reaction with hydroxylamine (0.2% v/v final concentration), labeled peptide samples were combined and dried by vacuum centrifugation.

### 2.6 Peptide fractionation

The combined TMT-labeled peptides were re-constituted in 100 μL 100 mM TEAB buffer and filtered through EasyPep Mini cleanup columns (Thermo Fisher Scientific) to remove excess TMT label and hydroxylamine. Peptides in the flow through were diluted to 2 mL in 10 mM TEAB in water and loaded on a XBridge C18 Guard Column (5 μm, 2.1 × 10 mm, Waters) at 250 μL/min for 8 min prior to fractionation on a XBridge C18 Column (5 μm, 2.1 × 100 mm column (Waters) using a 0–90% acetonitrile (ACN) in 10 mM TEAB gradient over 85 min at 250 μL/min on an Agilent 1200 series capillary HPLC with a micro-fraction collector. Eighty-four 250 μL fractions were collected and concatenated into 24 fractions according to the method described by Wang et al. and dried [30].

### 2.7 Liquid chromatography separation and tandem mass spectrometry (LC-MS/MS) for proteomics

Dried peptides were reconstituted in 2% ACN and 0.1% formic acid (FA) and analyzed by nanoflow liquid chromatography-tandem mass spectrometry (nLC-MS/MS) using a Neo Vanquish UHPLC interfaced with an Orbitrap Exploris 480 mass spectrometer (both instruments, Thermo Fisher Scientific). Peptide separation was performed with linear gradients (water/acetonitrile) over polyimide-coated, fused-silica, 25 cm × 360 μm o.d./75 μm i.d. self-packed columns with Kasil frits and PepSep stainless steel emitter (30 μm inner diameter, Bruker Daltonics). Stationary phase in analytical columns consisted of ReproSil-Pur 120C18-AQ, 2.4 μm particle size, 120 Å pore (Dr. Maisch High Performance LC GmbH). Trap columns consisted of ∼1 cm × 360 μm o.d./75 μm i.d. polyimide-coated, fused-silica tubing (New Objective), packed with 5 μm particle size, 120 Å pore, C18 stationary phase (ReproSil-Pur), with a Kasil frit. Electrospray ionization was accomplished with 2 kV positive spray voltage and an ion transfer tube temperature of 250 °C. For DDA TMT analysis, each peptide fraction was separated by a 120-minute linear gradient. MS1 precursor ion scans were acquired in the Orbitrap detector of the Exploris 480 mass spectrometer with a 3 s cycle time from 400 to 1500 *m*/*z* at 120,000 resolution at 200*m*/*z* with a normalized AGC of 300%, an RF lens setting of 50%, and maximum injection time set to Auto. Precursor ions were individually isolated with a 0.7*m*/*z* isolation window and a 3-second duty cycle, with the following filters: monoisotopic precursor selection (MIPS) set to peptide mode, intensity threshold 2.5 × 104, charge states 2–6, dynamic exclusion duration of 45 s (10 ppm tolerance), isolation purity 70%. Precursors were fragmented by HCD with a 36% normalized collision energy and MS/MS spectra were acquired at 30,000 resolution, AGC set to Standard and maximum injection time set to Auto.

### 2.8 Bulk proteomics data analysis

All database searches were done in Proteome Discoverer v3.1 (Thermo Fisher Scientific) with Chimerys using the Inferys v3.0 prediction model. For TMT analysis, all fractions were searched together against a human UniProt FASTA database (proteome accession UP000005640, 98758 entries) and a custom database containing common contaminants (e.g., human keratin; 438 entries). Search criteria were tryptic cleavage (maximum 2 missed), peptide length 7–30, peptide charge 2–6, 10 ppm fragment ion mass tolerance, Cys carbamidomethylation, Lys TMTpro labeling, and N-terminal TMTpro labeling as fixed modifications, and Met oxidation as a variable modification (max 3/peptide). Peptide identifications were validated by Chimerys at 1% false discovery rate (FDR) based on an auto-concatenated decoy database search. Peptide spectral matches (PSMs) were filtered for isolation interference ≤30% and a normalized Chimerys coefficient of 0.8. Only proteotypically unique peptides were used for relative quantitation. TMT reporter ions were normalized to total peptide amount per channel and quantification on reporter ion signal-to-noise values. Group differences were tested by ANOVA based on protein abundance with no imputation of missing values. Differential protein expression analysis, principal component analysis, gene set enrichment analysis, and plots were generated in RStudio with the following packages: limma, FactoMineR, EnhancedVolcano, clusterProfiler, factoextra, enrichplot, and ggplot2 [28,[31], [32], [33], [34], [35]]. Data outputs from these analyses are included in Source Data Figure 1, Source Data Figure 5, and Source Data Figure 6.

### 2.9 Extracellular flux measurements of metabolite concentrations in media

During [U–^13^C] glucose and [U–^13^C] glutamine tracing experiments, 300 μL of media was obtained for analysis every hour, flash frozen, and stored at −80 °C. For glucose, lactate, glutamine, and glutamate concentration measurements, media were input into a YSI 2900 Biochemistry Analyzer (YSI) equipped with Glucose/Lactate and Glutamine/Glutamate modules. Metabolite concentrations were converted to metabolite abundance and normalized to cell weight. The normalized metabolite abundances were used to determine the rate of change over time via linear regression analysis. The extracellular fluxes were plotted as nmol per milligram of cell weight per hour.

### 2.10 Isotope tracing with [U–^13^C] glucose, [1,2–^13^C] glucose, and [U–^13^C] glutamine

**Figure 2:**
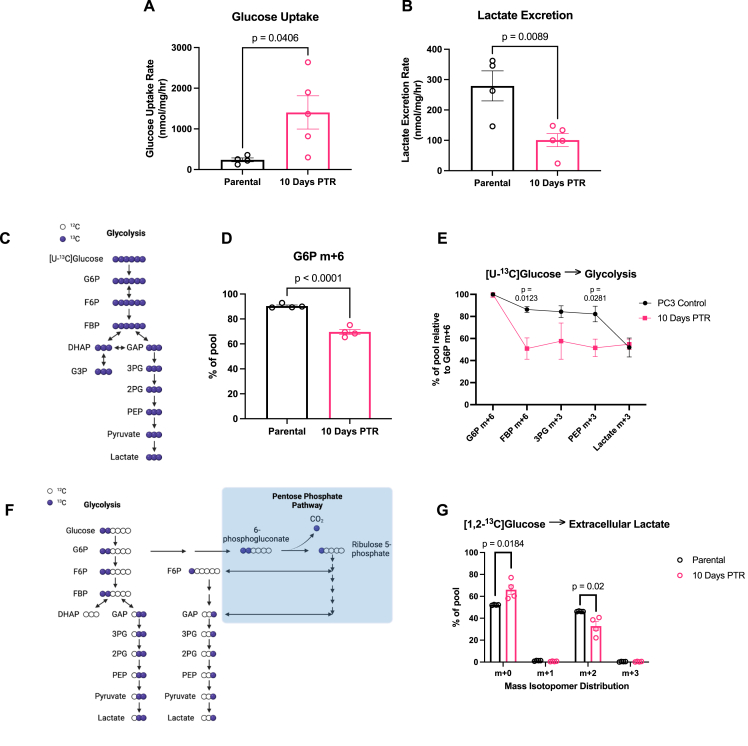
**Surviving cells increase glucose uptake and decrease flux through glycolysis**. (**A**) Glucose uptake rate of parental cells and cells 10 Days PTR. (**B**) Lactate excretion rate of parental cells and cells 10 Days PTR. (**C**) Schematic of ^13^C-atom transitions in the glycolytic pathway from [U–^13^C] glucose. Blue circles denote ^13^C. White circles denote ^12^C. Created in BioRender. Li, M. (2026) https://BioRender.com/vwr1a3f. (**D**) Labeling of intracellular G6P m+6 from [U–^13^C] glucose in parental cells and cells 10 Days PTR. (**E**) Labeling of intracellular glycolytic metabolites from [U–^13^C] glucose relative to intracellular G6P m+6 in parental cells and cells 10 Days PTR. (**F**) Schematic of ^13^C-atom transitions in the glycolytic pathway from [1,2–^13^C] glucose. Blue circles denote ^13^C. White circles denote ^12^C. Created in BioRender. Li, M. (2026) https://BioRender.com/hfybwb9. (**G**) Labeling of extracellular lactate m+2 from [1,2–^13^C] glucose from media of parental cells and cells 10 Days PTR. n = 4 biological replicates and n = 5 biological replicates for parental cells and cells 10 Days PTR, respectively (**A-B**); n = 4 biological replicates (**D-E, G**). Data are presented as mean ± s.e.m. P-values were calculated via unpaired Student's two-tailed t-test and exact values are indicated in the figure. Normal distributions were confirmed for all data with a Shapiro–Wilk test. Abbreviations: glucose 6-phosphate (G6P); fructose 1,6-bisphosphate (FBP); 3-phosphoglycerate (3 PG); phosphoenolpyruvate (PEP).

**Figure 3:**
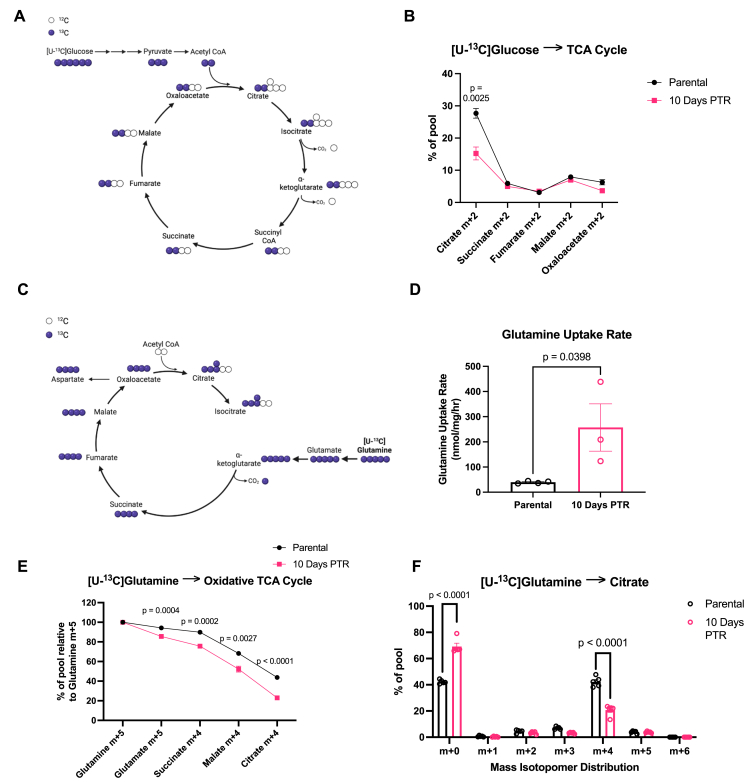
**Surviving cells decrease glutamine contribution into the TCA cycle**. (**A**) Schematic of ^13^C-atom transitions in the TCA cycle from [U–^13^C] glucose. Blue circles denote ^13^C. White circles denote ^12^C. Created in BioRender. Li, M. (2026) https://BioRender.com/ufaoeuo. (**B**) Labeling of intracellular TCA cycle metabolites from [U–^13^C] glucose in parental cells and cells 10 Days PTR. (**C**) Schematic of ^13^C-atom transitions in the TCA cycle from [U–^13^C] glutamine. Blue circles denote ^13^C. White circles denote ^12^C. Created in BioRender. Li, M. (2026) https://BioRender.com/ay5843y. (**D**) Glutamine uptake rate of parental cells and cells 10 Days PTR. (**E**) Labeling of intracellular TCA cycle metabolites from [U–^13^C] glutamine relative to intracellular glutamine m+5 in parental cells and cells 10 Days PTR. (**F**) Mass isotopomer distribution (MID) of citrate from [U–^13^C] glutamine in parental cells and cells 10 Days PTR. n = 4 biological replicates (**B**); n = 4 biological replicates and n = 3 biological replicates in parental cells and cells 10 Days PTR, respectively (**D**); n = 5 biological replicates (**E-F**). Data are presented as mean ± s.e.m. P-values were calculated via unpaired Student's two-tailed t-test and exact values are indicated in the figure. Normal distributions were confirmed for all data with a Shapiro–Wilk test.

Cells were cultured to the desired timepoints. On the day of the experiment, [U–^13^C] glucose (Cambridge Isotope Laboratories; Cat#: CLM-1396-10) or [1,2–^13^C] glucose (Cambridge Isotope Laboratories; Cat#: CLM-504-1) was added to RPMI 1640 Glucose-Free Media (Thermo Fisher Scientific; Cat# 11879020) at a final concentration of 11.11 mM with 10% dialyzed FBS (Thermo Fisher Scientific; Cat#: 26400044), and 1% Penicillin/Streptomycin (Gibco^TM^; Cat#15140-122). Cells were incubated with [U–^13^C] glucose or [1,2–^13^C] glucose tracer for 3 h and 6 h. At endpoint, cells were lifted with TrypLE^TM^ Express (Gibco^TM^; Ref# 12604-013), which was subsequently neutralized with dialyzed FBS-containing media and spun down at 500 g for 5 min at 4 °C. Cells were washed with 1X PBS twice and pelleted into a 1.5 mL Eppendorf tube. Cell weights were measured, and pellets were snap frozen with liquid nitrogen and stored at −80 °C until metabolite extraction. For [U–^13^C] glutamine experiments, [U–^13^C] glutamine (Cambridge Isotope Laboratories; Cat#: CLM-1822-0.5) was added to RPMI 1640 Glutamine-Free Media (Thermo Fisher Scientific; Cat# 11879020) at a final concentration of 2.20 mM with 10% dialyzed FBS (Thermo Fisher Scientific; Cat#: 26400044), and 1% Penicillin/Streptomycin (Gibco^TM^; Cat#15140-122). Cells were incubated with [U–^13^C] glutamine tracer for 3 h and 6 h. At endpoint, cells were lifted with TrypLE^TM^ Express (Gibco^TM^; Ref# 12604-013), which was subsequently neutralized with dialyzed FBS-containing media and spun down at 500 g for 5 min at 4 °C. Cells were washed with 1X PBS twice and pelleted into a 1.5 mL Eppendorf tube. Cell weights were measured, and pellets were snap frozen with liquid nitrogen and stored at −80 °C until metabolite extraction. Cells underwent freeze thaw cycles in liquid nitrogen followed by homogenization in 1:1 methanol:water using needle sonication. The samples were then centrifuged at 5,000 RPM for 10 min at 4 °C. Samples were filtered using a 3 kDa Amicon filter to remove proteins and lipids, dried using a speed vacuum, and then reconstitutied in a 1:1 methanol:water mixture. Glycolytic intermediates and tricarboxylic acid (TCA) cycle metabolites and their isotopomers were quantified using a Luna NH2 column (3 μm, 150 × 2 mm, Phenomenex, Torrance, CA), following previously described detailed methods [[36], [37], [38], [39]]. Natural abundance correction was performed with IsocorrectoR [40] and mass isotopomer distributions (MIDs) were plotted in GraphPad Prism as “% of pool”, indicating the relative amount of a specific mass isotopomer divided by the sum of all isotopomers of the metabolite and multiplied by 100. Data plotted in GraphPad Prism are included in Source Data Figure 2 and Source Data Figure 3.

### 2.11 Quantification of [1,2–^13^C] glucose contribution to lactate

Sample preparation was performed based on a protocol from Ahn & Antoniewicz [41]. Briefly, media samples were dried under nitrogen gas flow at 37 °C. The dried samples were then resuspended in 2% methoxylamine hydrochloride in pyridine and subsequently heated at 37 °C. Derivatization was performed with the addition of N-methyl-N-(tert-butyldimethylsiyl)-trifluoroacetamide (MTBSTFA) + 1% tertbutyldimethylchlorosilane (TBDMCS) and incubation at 60 °C. Samples were spun down at max speed for 5 min and supernatant was transferred to gas chromatography vials for GC–MS. GC–MS was performed using the Agilent 7890A GC connected to an Agilent 5977B Mass Spectrometer. Peak calling and integration were performed using PIRAMID [42].

### 2.12 ^13^C-metabolic flux analysis (^13^C-MFA)

**Figure 4:**
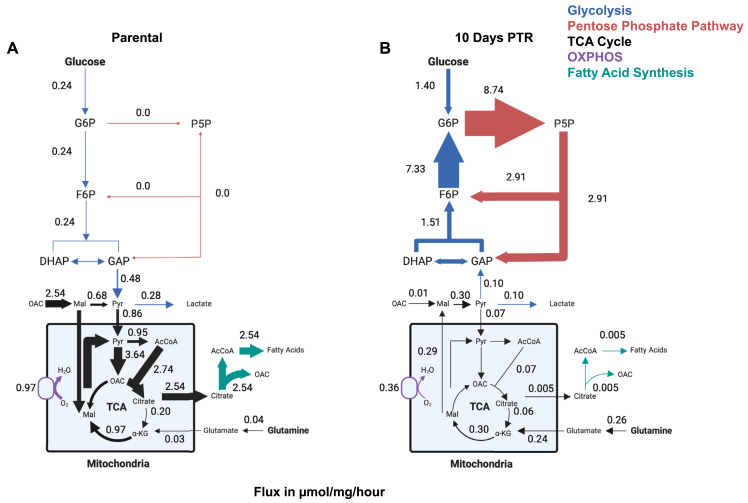
**^13^C-metabolic flux analysis reveals the reallocation of glucose and glutamine to fuel the pentose phosphate pathway in surviving cells**. (**A**) Flux map of central carbon metabolism (in units of μmol per milligram of cell weight per hour) in parental cells. Relative thickness of arrows corresponds to the magnitude of flux. Created in BioRender. Li, M. (2026) https://BioRender.com/r764b3m. (**B**) Flux map of central carbon metabolism (in units of μmol per milligram of cell weight per hour) in cells 10 Days PTR. Relative thickness of arrows corresponds to the magnitude of flux. Created in BioRender. Li, M. (2026) https://BioRender.com/mrphs8q. Numbers are best-fit flux values from ^13^C-metabolic flux analysis (^13^C-MFA) using isotopomer network compartmental analysis (INCA)^29^, constrained by metabolite ^13^C labeling from [U–^13^C] glucose and [U–^13^C] glutamine experiments for both parental cells and cells 10 Days PTR, each with n = 5 biological replicates, and metabolite uptake and excretion rates (n = 3–5 biological replicates).

**Figure 5:**
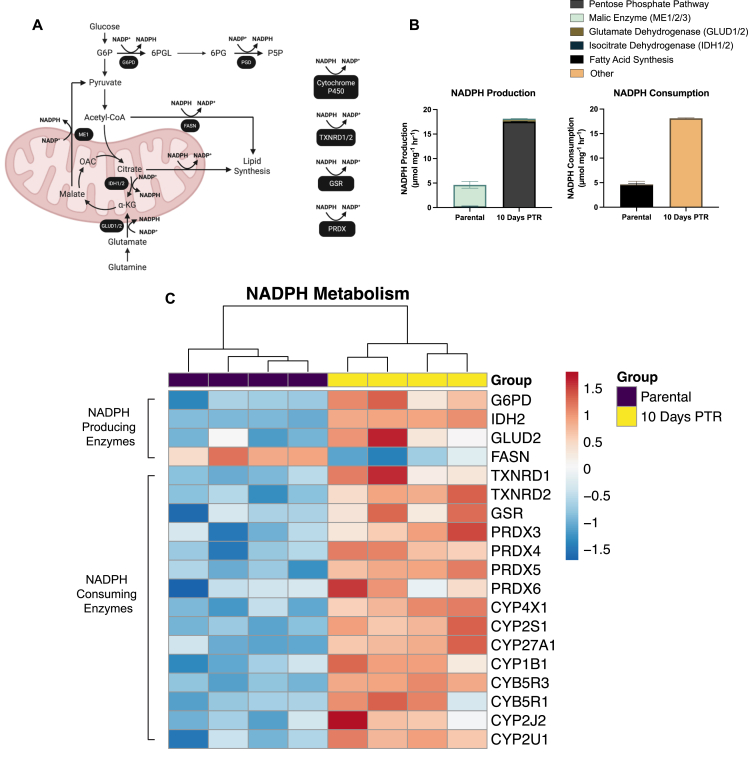
**Reprogramming of central carbon metabolism increases NADPH production and utilization in surviving cells.** (**A**) Schematic of key NADPH-producing and consuming reactions in central carbon metabolism. Enzymes that mediate each reaction are labeled in under the reaction arrow. Created in BioRender. Li, M. (2026) https://BioRender.com/kitiup9. (**B**) NADPH-producing and consuming fluxes in parental cells and cells 10 Days PTR, calculated stoichiometrically from the best-fit flux values from ^13^C-metabolic flux analysis. Relative contribution of pathways to NADPH production and consumption, such as the pentose phosphate pathway, malic enzyme (ME1/2), glutamate dehydrogenase (GLUD1/2), isocitrate dehydrogenase (IDH1/2), fatty acid synthase, and central carbon metabolism-independent reactions, were calculated and compared between parental cells and cells 10 Days PTR. (**C**) Heatmap of differentially expressed proteins that drive NADPH production and consumption in parental cells and cells 10 Days PTR. n = 4 biological replicates (**C**). Data are presented as mean ± s.e.m. (**B**).

Metabolic flux analysis was performed with the INCA 2.4 software based on the elementary metabolite unit (EMU) framework [43,44]. Mass isotopomer distributions (MIDs) of measured metabolites from [U–^13^C] glucose and [U–^13^C] glutamine tracer experiments and measured extracellular fluxes (glucose and glutamine import, lactate export, and oxygen consumption) were integrated into a defined isotopomer network model of central carbon metabolism. The oxygen consumption flux was determined previously by our group [15]. Fluxes were estimated by minimizing the sum of squared residuals (SSR) between simulated and observed MIDs [43,44]. Best-fit flux values were determined through the least squares regression of 100 random initial guesses. A chi-squared test was performed to assess goodness-of-fit of the flux solutions, and parameter continuation was performed to obtain 95% confidence intervals for each calculated flux [43]. Model fits for both parental cells and cells 10 Days PTR were accepted with SSRs within their respective 95% confidence intervals. Data outputs from INCA 2.4 are included in Source Data Figure 4. Data outputs of NADPH flux calculations are included in Source Data Figure 5.

### 2.13 Genome scale metabolic modeling

Genome scale metabolic modeling using the Recon3D metabolic network was performed through Constraint-Based Optimization and Reconstruction Analysis (COBRA) in MATLAB (MathWorks) [45,46]. Proteomic integration into the model was performed based on the E-Flux method, where empirical protein expression data was used to set upper and lower bounds for reaction fluxes based on the assumption that higher protein expression leads to higher flux [47]. log_2_-transformed proteomics expression data was globally normalized so that the highest expression value was equal to 1. These normalized expression values were used as coefficients to set flux bounds for their corresponding reactions. The default maximum flux for each reaction was set to 10,000 nmol/mg/hr. The maximum flux of a certain reaction after proteomic integration was the default value multiplied by the expression coefficient. Flux data from ^13^C-MFA were used to set constraints on corresponding reactions in the reduced Recon3D model. No objective function was set. Coordinate hit-and-run with rounding Markov-Chain Monte Carlo (CHRR-MCMC) analysis was performed to sample flux distributions from the constrained metabolic models of cells 10 Days PTR and parental cells [48]. For each model, the flux space was defined based on the proteomic and ^13^C-MFA constraints. Flux sampling was performed on 1000 samples, where the number of skips between samples was set to 100. P-values were calculated using bootstrapped Mann–Whitney U tests. For each of the 100 bootstrap iterations, 100 samples were randomly selected from each group, and a Mann–Whitney U test was performed. The median p-value for each reaction is indicated in the figure. Raw data output from CHRR-MCMC sampling are included in Source Data Figure 7, Source Data Supplementary Fig. 1, and Source Data Supplementary Fig. 2. Metabolic maps were visualized in Escher [49]. Escher maps and corresponding data input from CHRR-MCMC sampling are included in Supplementary Data 1–3 to visualize metabolic fluxes at https://escher.github.io/. Nomenclature of all the reactions from genome scale modeling is from the Recon3D model reconstruction [46].

### 2.14 Pentose phosphate pathway & antioxidant gene set scoring and survival analysis

Patient RNA expression data was downloaded from publicly available datasets from cBioPortal (https://www.cbioportal.org). Transcripts per million (TPM) or fragments per kilobase million (FPKM) expression values were normalized by log_2_(TPM +1) and log_2_(FPKM +1), respectively, and subsequently used for analysis. The datasets used in this paper are listed as “Metastatic Prostate Adenocarcinoma (SU2C/PCF Dream Team, PNAS 2019)”, “Breast Invasive Carcinoma (TCGA, Firehose Legacy)”, and “Liver Hepatocellular Carcinoma (TCGA, Firehose Legacy)”. The Pentose Phosphate Pathway (PPP)-Antioxidant gene set used for analysis was defined through the WikiPathways “Pentose Phosphate Metabolism” and “NRF2 Pathway” gene sets [50]. It consisted of the following genes: *G6pd*, *Pgd*, *Pgls*, *Tkt*, *Taldo1*, *Rpe*, *Rpia*, *Nfe2l2*, *Gclc*, *Gclm*, *Gpx2*, *Gpx3*, *Gsr*, *Prdx1*, *Prdx6*, *Slc7a11*, *Sod3*, *Txn*, *Txnrd1*, *Txnrd3*. To calculate a PPP-Antioxidant expression score for each patient from each dataset, gene set variation analysis (GSVA) was performed on the log_2_-transformed TPM or FPKM expression of the indicated genes [51]. Within each dataset, patients in the upper quartile (≥75th percentile) of PPP-Antioxidant expression scores were classified as “High”, while those in the lower quartile (≤25th percentile) were classified as “Low”. Kaplan–Meier survival and univariate Cox regression analyses assessed the impact of PPP-Antioxidant expression on overall survival. Analyses were performed in R using the survival and survminer packages [52,53]. Data outputs from these analyses are included in Source Data Figure 8.

### 2.15 Statistics

Statistical analyses were performed in GraphPad Prism version 9.1 (GraphPad Software, LLC) and R. Statistical tests that were performed and p-values are reported in the figures and figure legends. An ⍺ value of 0.05 was used for all tests.

## 3 Results

### 3.1 Cells that survive cisplatin treatment exhibit transcriptomic and proteomic signatures of increased glucose metabolism and decreased oxidation phosphorylation

Prostate cancer PC3 parental cells were treated with an LD_50_ dose of cisplatin for 72 h, treatment was removed, and cultures were maintained to 10 days post-treatment removal (10 Days PTR). Throughout that 10-day period, cells presented with a non-proliferative phenotype and a continuous increase in cell size (Figure 1A). We have previously shown that cells continue to die days after treatment removal, with the death rate plateauing at 10 days [3]. Time-resolved transcriptomic profiling through single-cell RNA sequencing (scRNASeq) revealed an increase in glycolysis-related gene expression and a decrease in oxidative phosphorylation (OXPHOS)-related gene expression relative to parental as cells progressed from 1 Day PTR to 10 Days PTR (Figure 1B). In addition, the percentage of cells exhibiting changes in glycolytic gene expression increased over time from 1 Day PTR to 10 Days PTR, while the percentage of cells exhibiting changes in OXPHOS gene expression remained mostly the same (Figure 1B). These data suggest that cells progressively adopt glycolytic states to survive following treatment removal.

To assess if the transcriptomic changes are also observed at the protein level, we profiled proteomic differences between cells 10 Days PTR and parental cells. Overall, 9,277 proteins were detected. 6,125 differentially expressed proteins were identified between sample groups, with 2,451 upregulated proteins in cells 10 Days PTR and 3,674 upregulated proteins in parental cells (Figure 1C). Consistent with the observed gene expression changes from scRNA-seq, key proteins mediating glycolysis and gluconeogenesis such as SLC2A1, ENO2, FBP1, and PCK2 were increased in cells 10 Days PTR, while proteins mediating OXPHOS such as COX5B, COX14, and NDUFS6 were decreased (Figure 1C). These findings were further supported by the enrichment of the Hallmark Glycolysis gene set and a depletion in the WikiPathways Oxidative Phosphorylation gene set in cells 10 Days PTR via pathway enrichment analysis (Figure 1D–E). Based on these data, we hypothesized that cells 10 Days PTR increase glucose uptake and production while shifting mitochondrial metabolism away from ATP production.

### 3.2 Surviving cells increase glucose uptake and decrease flux through glycolysis

Given the enrichment of genes and proteins mediating glycolysis and gluconeogenesis in cells 10 Days PTR, we next investigated how glucose is metabolized in cells 10 Days PTR and in parental cells. Glucose is imported into the cell and typically metabolized to pyruvate. As described by the Warburg Effect, cancer cells utilize pyruvate to generate lactate, releasing it into the extracellular space at high concentrations [54]. We measured the extracellular fluxes of glucose uptake and lactate excretion and found that cells 10 Days PTR had a 5.9-fold increase in glucose uptake rate compared to parental cells (Figure 2A). Despite the increase in glucose uptake rate, cells 10 Days PTR excreted less lactate over time (Figure 2B). To investigate how glucose is metabolized intracellularly, we cultured cells in media containing [U–^13^C] glucose and dialyzed FBS and analyzed the contribution of the isotope tracer to downstream glycolytic metabolites (Figure 2C). While we observed increased glucose import in cells 10 Days PTR, we found a reduction in [U–^13^C] glucose contribution to glucose 6-phosphate (G6P) when compared to parental cells (Figure 2D). This was perplexing since G6P is the first metabolite downstream of glucose import, where glucose gets phosphorylated at the C6-position [55]. This suggests that there may be another carbon source contributing to the G6P pool. One possible mechanism is through phosphoglucose isomerase (PGI), which can reversibly generate G6P from fructose 6-phosphate (F6P) [56]. We also observed a dramatic drop in contribution of [U–^13^C] glucose to the fructose 1,6-bisphosphate (FBP) pool, suggesting that the tracer is diluted by an unlabeled carbon source (Figure 2E). The contribution of [U–^13^C] glucose to the other downstream glycolytic metabolites was decreased in cells 10 Days PTR when compared to parental cells (Figure 2E). We measured tracer incorporation into extracellular lactate with [1,2–^13^C] glucose using gas chromatography mass spectrometry (GC–MS) and observed decreased levels of m+2 lactate in the media of cells 10 Days PTR. Taken together, these data demonstrate that surviving cells decrease glucose contribution to glycolysis and reduce glycolytic flux (Figure 2F–G).

### 3.3 Glutamine is the major carbon source for the TCA cycle in parental cells and surviving cells

Downstream from glycolysis, pyruvate is oxidized to acetyl-CoA, enters the mitochondria, and metabolized through the TCA cycle [57]. Through [U–^13^C] glucose tracing, we found that cells 10 Days PTR exhibit decreased labeling of m+2 citrate, suggesting decreased glucose-derived influx into the TCA cycle when compared to parental cells (Figure 3A–B). In both parental cells and cells 10 Days PTR, labeling fractions of metabolites downstream of citrate decreased dramatically to under 10%, indicating that the [U–^13^C] glucose tracer was diluted by an unlabeled carbon source (Figure 3B). The dilution of the [U–^13^C] glucose tracer can be first observed in the labeling of m+2 succinate. Succinate is downstream of alpha-ketoglutarate, which is a key node where glutamine-derived carbons can enter the TCA cycle. It has been shown in various studies that glutamine is another major source of carbon for the TCA cycle in cancer cells [[58], [59], [60]], so we hypothesized that both parental cells and cells 10 Days PTR were utilizing glutamine to fuel the TCA cycle instead of glucose.

### 3.4 Surviving cells decrease glutamine contribution into the TCA cycle

We measured the contribution of [U–^13^C] glutamine to TCA cycle metabolites (Figure 3C). Carbons from [U–^13^C] glutamine incorporate into glutamate via glutaminase (GLS) activity to generate glutamate m+5, followed by its conversion to alpha-ketoglutarate (⍺-KG) m+5 through glutamate dehydrogenase (GDH) (Figure 3C). ⍺-KG is then decarboxylated and leads to m+4 labeling of the downstream TCA cycle metabolites such as succinate, fumarate, malate, and oxaloacetate (Figure 3C). m+4 citrate is generated as m+4 oxaloacetate reacts with unlabeled acetyl-CoA (Figure 3C). Cells were cultured in [U–^13^C] glutamine-containing media and media was sampled every hour for analysis of the extracellular rate of glutamine uptake. Cells 10 Days PTR exhibited increased glutamine consumption over time when compared to parental cells (Figure 3D). However, we observed that despite the increase in glutamine uptake rate, cells 10 Days PTR show decreased [U–^13^C] glutamine contribution to the intracellular TCA cycle metabolites compared to parental cells (Figure 3E). For example, there was decreased enrichment of m+4 citrate from [U–^13^C] glutamine in cells 10 Days PTR, suggesting that other carbon sources are contributing to the citrate pool (Figure 3F). In addition, labeling of m+5 citrate from [U–^13^C] glutamine was lower than 10% in both sample groups, indicating that glutamine does not fuel reductive carboxylation (Figure 3F).

### 3.5 ^13^C-metabolic flux analysis reveals the reallocation of glucose and glutamine to fuel the pentose phosphate pathway in surviving cells

One limitation of solely using mass isotopomer distributions (MIDs) to assess intracellular metabolic activity is the inability to report absolute fluxes of reactions in a pathway. MID data allows us to make conclusions on the relative pathway contributions to the production of a metabolite and therefore can only provide qualitative information [61,62]. To quantify the differences in absolute fluxes of metabolic reactions between parental cells and cells 10 Days PTR, we employed computational modeling to perform ^13^C-metabolic flux analysis (^13^C-MFA) based on established methods by integrating the MID data from the [U–^13^C] glucose and [U–^13^C] glutamine experiments into a defined metabolic model and constraining the model using the measured extracellular fluxes [43,63].

^13^C-MFA revealed that parental cells primarily metabolized glucose via glycolysis, with glucose-derived pyruvate and lactate production at 0.48 μmol/mg/hr and at 0.28 μmol/mg/hr, respectively (Figure 4A). These fluxes are consistent with other reports of glycolytic flux in proliferating cancer cells [44,64,65]. In contrast, cells 10 Days PTR metabolized glucose by shuttling G6P into the oxidative pentose phosphate pathway (oxPPP) at a rate of 8.74 μmol/mg/hr, resulting in the production of pentose 5-phosphate (P5P) (Figure 4B). Cells 10 Days PTR also exhibit high flux through the non-oxidative pentose phosphate pathway (non-oxPPP), feeding carbons from P5P back into the glycolytic pathway through the generation of fructose 6-phosphate (F6P) and glyceraldehyde 3-phosphate (GAP) (Figure 4B). Interestingly, the net fluxes of the glycolytic reactions in cells 10 Days PTR indicate that glycolysis is operating in the reverse direction, suggesting that in cells 10 Days PTR activate gluconeogenesis with carbons derived from pyruvate (Figure 4B).

Mitochondrial metabolism of pyruvate was highly active in parental cells through pyruvate dehydrogenase, generating acetyl-CoA at 0.95 μmol/mg/hr, and pyruvate carboxylase, generating oxaloacetate (OAC) at 3.64 μmol/mg/hr (Figure 4A). Downstream of OAC generation, citrate is synthesized and mostly exported out of the mitochondria in parental cells for fatty acid synthesis, producing cytosolic OAC as a byproduct (Figure 4A). In cells 10 Days PTR, we observed decreased pyruvate import into the mitochondria and a dramatic decrease in citrate export for fatty acid synthesis (Figure 4B). Although cells 10 Days PTR mainly oxidize citrate to alpha-ketoglutarate in the TCA cycle, the flux of that reaction is over 3-fold lower than in parental cells (Figure 4A–B).

Glutamine is another carbon source that feeds into the TCA cycle downstream of citrate. Cells 10 Days PTR take up glutamine at a higher rate than parental cells and ultimately synthesize mitochondrial malate in the TCA cycle. Interestingly, cells 10 Days PTR export malate into the cytosol and convert it to pyruvate, which would then feed carbons into the gluconeogenic pathway (Figure 4B). In contrast to cells 10 Days PTR, parental cells retain TCA cycle-derived malate in the mitochondria (Figure 4A). The consumption of TCA cycle metabolites to support gluconeogenesis in cells 10 Days PTR is one possible explanation for the decreased relative pool sizes of intracellular glutamine, glutamate, succinate, and aspartate (Supplementary Fig. 1A–G). These data suggest that cells 10 Days PTR metabolize glutamine through the TCA cycle with an anaplerotic function to provide carbons for gluconeogenesis. We hypothesize that the G6P generated from this process and from glucose phosphorylation are then utilized to fuel the oxPPP.

### 3.6 Reprogramming of central carbon metabolism increases NADPH production and utilization in surviving cells

One of the main functions of the oxPPP is to generate a key reducing molecule, NADPH [66]. It can be utilized to reduce oxidized glutathione and replenish reduced thioredoxins for antioxidant defense, consumed for de novo fatty acid synthesis, or used by cytochrome P450 (CYP450) enzymes to metabolize xenobiotics [[66], [67], [68]]. Using the quantified metabolic fluxes from our ^13^C-MFA modeling, we estimated NADPH production and consumption fluxes based on known reaction stoichiometry. For example, the flux of NADPH consumption for de novo fatty acid synthesis is equal to the fatty acid synthesis carbon flux multiplied by a factor of 1.75, as 14 molecules of NADPH and 8 molecules of acetyl-CoA are consumed to generate one molecule of palmitate [67].

To systematically evaluate NADPH sources in cisplatin-surviving cells, we quantified fluxes of all major central carbon metabolism-related NADPH-generating reactions from our metabolic model, including the PPP, malic enzyme (ME), glutamate dehydrogenase (GLUD1/2), and isocitrate dehydrogenase (IDH1/2) (Figure 5A). We first calculated the sum of all the fluxes that contributed to NADPH production in each group and subsequently assessed the relative contribution of those reactions to the total NADPH production flux. Cells 10 Days PTR exhibited a 3.9-fold increase in total NADPH production based on the reactions described above (Figure 5B). Nearly all of the NADPH produced in PC3 parental cells is through cytosolic and mitochondrial malic enzymes (ME1/2), which catalyze the conversion of malate to pyruvate, with a combined flux of 4.14 μmol/mg/hr (Figure 5A–B). On the other hand, nearly all of the NADPH produced in cells 10 Days PTR is through the oxPPP via glucose 6-phosphate dehydrogenase (G6PD) and phosphogluconate dehydrogenase (PGD) at a combined flux of 17.50 μmol/mg/hr (Figure 5A–B). This data indicates that not only is NADPH production increased in cells 10 Days PTR, but that the oxPPP is the dominant contributor of NADPH in cells 10 Days PTR rather than ME1/2 as observed in parental cells. Based on the metabolic steady state assumption and law of mass balance used in ^13^C-MFA, NADPH production must equal NADPH consumption, allowing us to infer that cells 10 Days PTR also exhibit a 3.9-fold increase in total NADPH consumption when compared to parental cells (Figure 5B) [44,67]. Nearly all of the NADPH consumed in parental cells is through fatty acid synthesis, while reactions that were not captured by our core metabolic model, labeled “Other”, contributed the most to NADPH consumption in cells 10 Days PTR (Figure 5B). These reactions include thioredoxin reductase (TXNRD1/2), glutathione reductase (GSR), and peroxiredoxin (PRDX), which are key components in antioxidant defense, and CYP450 (Figure 5A) [69]. Furthermore, cells 10 Days PTR exhibit increased protein expression of NADPH-producing enzymes (G6PD, IDH2, and GLUD2) and antioxidant metabolism enzymes that consume NADPH (Figure 5C).

**Figure 6:**
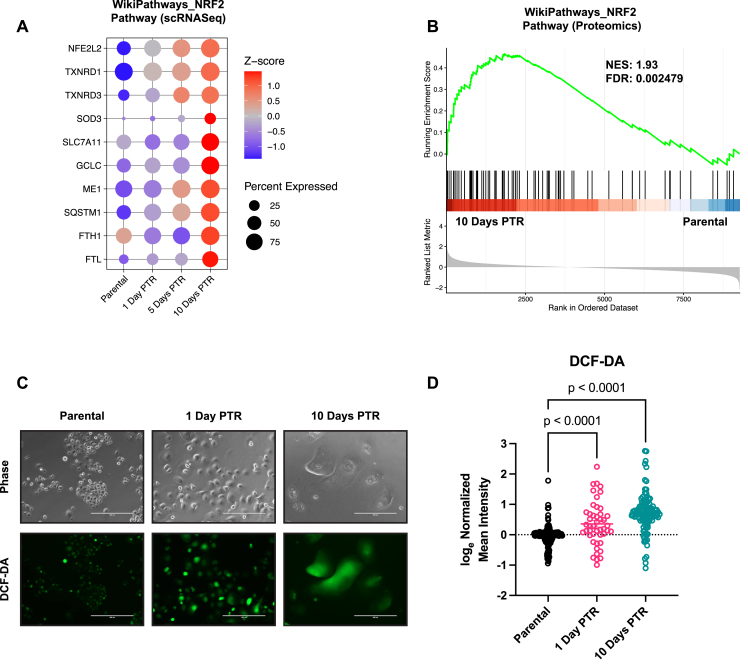
**Surviving cells demonstrate increased ROS levels and progressive NRF2 activation**. (**A**) Single-cell RNA expression bubble plots of leading-edge genes from Wikipathways NRF2 Pathway gene set. (**B**) WikiPathways NRF2 Pathway proteomic pathway enrichment analysis between cells 10 Days PTR and parental cells. (**C**) Representative phase contrast and DCF-DA images of parental cells and cells 1- and 10 Days PTR. (**D**) Normalized log_e_ mean intensity quantifications of DCF-DA staining between parental cells and cells 1- and 10 Days PTR. n = 3 biological replicates for parental cells and cells 1-, 5-, and 10 Days PTR (**A**). n = 4 biological replicates for parental cells and cells 10 Days PTR (**B**). n_Parental_= 176 cells, n_1 Day PTR_= 46 cells, n_10 Days PTR_= 105 cells (**C-D**).

G6PD and the majority of the antioxidant enzymes that were overexpressed in cells 10 Days PTR are regulated through nuclear factor erythroid 2-related factor 2 (NRF2) signaling [70]. Time-resolved transcriptomic profiling reveals an enrichment of NRF2 target gene expression relative to parental as cells progress from 1 Day PTR to 10 Days PTR, with the highest expression levels and percentage of cells expressing these genes observed at 10 Days PTR (Figure 6A). Pathway enrichment analysis from our proteomics dataset confirmed NRF2 pathway upregulation at the protein level in cells 10 Days PTR relative to parental cells (Figure 6B). We then hypothesized that the progressive activation of NRF2 in surviving cells is due to sustained oxidative stress throughout the period after treatment removal. We observed through DCF-DA staining that levels of reactive oxygen species (ROS) increased over time from 1 Day PTR to 10 Days PTR, relative to parental cells (Figure 6C–D). Taken together, these data suggest that cells 10 Days PTR increase NADPH production through the oxPPP and utilize it to fuel antioxidant metabolism to combat oxidative stress.

### 3.7 Surviving cells exhibit an increase in antioxidant metabolism and sustain antioxidant activity following in silico knockout of the oxidative pentose phosphate pathway

**Figure 7:**
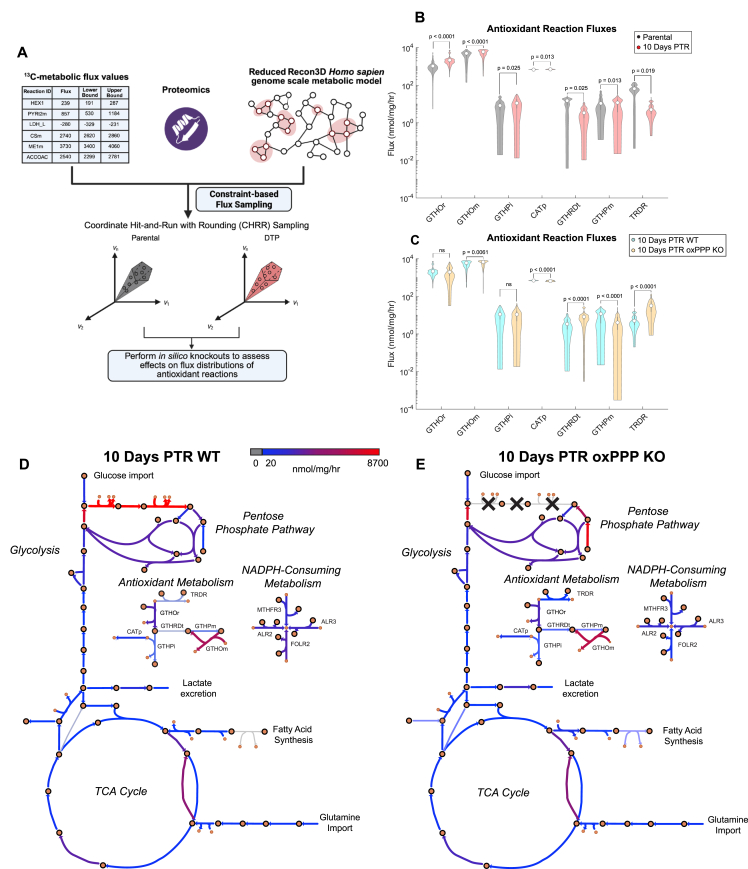
**Surviving cells exhibit an increase in antioxidant metabolism and sustain antioxidant activity following *in silico* knockout of the oxidative pentose phosphate pathway**. (**A**) Schematic of genome scale metabolic modeling workflow. Created in BioRender. Li, M. (2026) https://BioRender.com/8fsa6h0. (**B**) Sampled flux estimates of key antioxidant reactions between parental cells and cells 10 Days PTR (in units of nmol/mg/hr). (**C**) Sampled flux estimates of key antioxidant reactions between cells 10 Days PTR wild-type (WT) and cells 10 Days PTR oxPPP knockout (KO) (in units of nmol/mg/hr). (**D**) Escher map of cells 10 Days PTR WT condition. (**E**) Escher map of cells 10 Days PTR oxPPP KO condition. Large circles with black outlines represent metabolites, and small circles represent NADPH. Color of arrows correspond to flux value, ranging from 0 to 8700 nmol/mg/hr. n = 1000 CHRR samples per reaction, with 100 skips per CHRR sample (**B–C**). Data are presented as median and interquartile range (**B–C**). P-values were calculated using bootstrapped Mann–Whitney U tests. The median p-value for each reaction is indicated in figure **(B–C**). Abbreviations: cytosolic glutathione oxidoreductase (GTHOr), mitochondrial glutathione oxidoreductase (GTHOm), cytosolic glutathione peroxidase (GTHPi), catalase (CATp), glutathione transport to mitochondria (GTHRDt), mitochondrial glutathione peroxidase (GTHPm), thioredoxin reductase (TRDR).

To assess antioxidant reaction fluxes in cells 10 Days PTR and parental cells, we expanded our metabolic model to the genome scale with the Recon3D metabolic network reconstruction [46]. Our ^13^C-MFA data was limited to only central carbon metabolism, containing 48 essential reactions whereas Recon3D contains 13,543 [46]. To better reflect the metabolic state of our cells, the Recon3D model was first reduced to only contain reactions observed in our ^13^C-MFA and bulk proteomics datasets, resulting in a network of 6,039 reactions (Supplementary Table 1). We integrated our bulk proteomic expression data and ^13^C-MFA reaction fluxes as constraints in the reduced Recon3D model and performed constraint-based sampling to estimate fluxes of key NADPH-mediated and antioxidant reactions (Figure 7A and Supplementary Table 2). Relative to parental cells, cells 10 Days PTR exhibit increased fluxes of various antioxidant reactions such as cytosolic glutathione oxidoreductase (GTHOr), mitochondrial glutathione oxidoreductase (GTHOm), glutathione peroxidase (GTHPi), and catalase (CATp). In contrast, cells 10 Days PTR reduce flux through thioredoxin reductase (TRDR) (Figure 7B).

One powerful advantage of genome scale metabolic modeling is the ability to perform in silico knockouts of specific reactions and assess the effects on other reaction fluxes [45]. We simulated a knockout of the oxPPP in cells 10 Days PTR by setting the G6PD and PGD fluxes to zero and observed an increase in mitochondrial glutathione transport (GTHRDt), mitochondrial glutathione oxidoreductase (GTHOm), and TRDR fluxes, and a decrease in CATp flux (Figure 7C). Interestingly, we observed no changes in GTHOr (Figure 7C). These data suggest that antioxidant metabolism is sustained in response to depletion of oxPPP-derived NADPH. We then hypothesized that cells 10 Days PTR may be altering other NADPH-mediated metabolic reactions in response to oxPPP knockout to balance NADPH pools for antioxidant activity. We observed an increase in NADPH-producing reaction fluxes such as methylenetetrahydrofolate dehydrogenase (MTHFD) and malic enzyme (ME2) in cells 10 Days PTR oxPPP knockout when compared to wild-type (Supplementary Fig. 2A). Notably, cells 10 Days PTR under oxPPP knockout conditions exhibited decreased flux through NADPH-consuming reactions associated with folate metabolism (Figure 7D–E and Supplementary Fig. 2B). Folate reductase (FOLR2) experienced a 5.40-fold decrease and 5,10-methylenetetrahydrofolate reductase (MTHFR3) experienced a 1.50-fold decrease in flux (Figure 7D–E and Supplementary Fig. 2B). These data suggest that cells 10 Days PTR reduce cytosolic folate metabolism to compensate for the decrease in NADPH production, highlighting their potential metabolic flexibility to retain antioxidant metabolism in response to oxPPP inhibition.

### 3.8 High expression of pentose phosphate pathway and antioxidant genes is associated with poor prognosis across multiple cancer types

**Figure 8:**
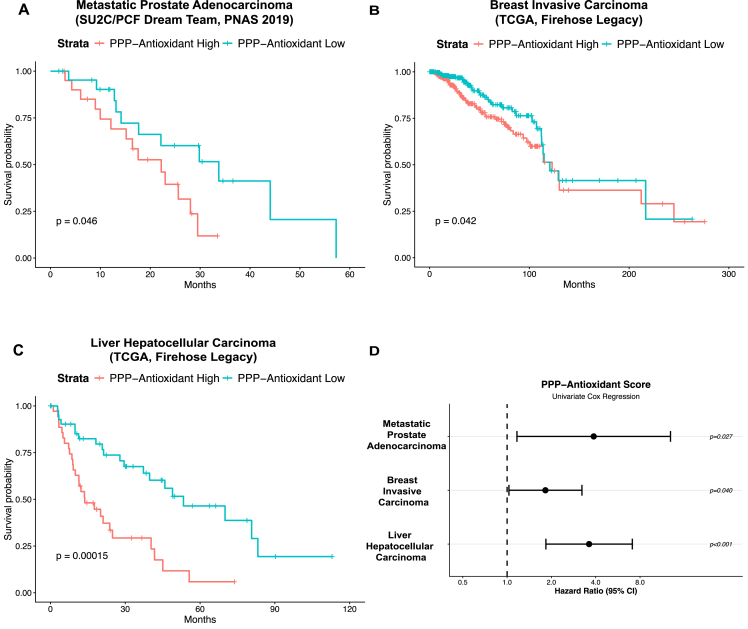
**High expression of pentose phosphate pathway and antioxidant genes is associated with poor prognosis across multiple cancer types**. (**A**) Kaplan–Meier survival analysis of patients stratified by PPP-Antioxidant High and PPP-Antioxidant Low from the Metastatic Prostate Adenocarcinoma (SU2C/PCF Dream Team, PNAS 2019) dataset. (**B**) Kaplan–Meier survival analysis of patients stratified by PPP-Antioxidant High and PPP-Antioxidant Low from the Breast Invasive Carcinoma (TGCA, Firehose Legacy) dataset. (**C**) Kaplan–Meier survival analysis of patients stratified by PPP-Antioxidant High and PPP-Antioxidant Low from the Liver Hepatocellular Carcinoma (TGCA, Firehose Legacy) dataset. (**D**) Univariate Cox regression analysis of the impact of PPP-Antioxidant expression on overall survival.

To assess the clinical relevance of pentose phosphate pathway (PPP) and antioxidant metabolism, we investigated the expression of key PPP and antioxidant genes from publicly available patient datasets and analyzed their prognostic value. We selected prostate cancer, breast cancer, and liver cancer datasets as polyploidy, a key feature of the resistant cell state we observe in surviving cells after cisplatin treatment, is frequently documented in these diseases and associated with metastatic progression and poor survival [6,10,11,13,14,[71], [72], [73]]. Within each dataset, we calculated a PPP-Antioxidant gene set expression score for each patient and stratified patients into “PPP-Antioxidant High” and “PPP-Antioxidant Low” groups. Through Kaplan–Meier survival analysis and univariate Cox regression analysis, we identified that high PPP-Antioxidant expression is associated with poor survival when compared to low PPP-Antioxidant expression in metastatic prostate cancer patients and in patients with breast invasive carcinoma and liver hepatocellular carcinoma (Figure 8A–D). These findings demonstrate that the metabolic adaptations we observed in cisplatin-surviving polyploid cells are clinically relevant and associated with poor patient outcomes in multiple cancer types.

## 4 Discussion

Our study reveals systems-level metabolic shifts that occur in PC3 cells that survive cisplatin treatment. A central finding of our work is that surviving cells rewire central carbon sources to prioritize NADPH production. ^13^C-MFA modeling revealed that cells 10 Days PTR increase glucose uptake and shuttle it into the oxPPP (Figure 4A–B). Additionally, cells 10 Days PTR export glutamine-derived carbons out of the TCA cycle to support gluconeogenesis, resulting in the production of G6P that can also fuel the oxPPP (Figure 4B). These data are consistent with the diluted G6P labeling from [U–^13^C] glucose observed in cells 10 Days PTR despite having an increase in glucose consumption rate (Figure 2A,D), as carbons from unlabeled glutamine are utilized anaplerotically to generate G6P via gluconeogenesis. Future work to validate these observations involves culturing cells in glutamine-deprived media or treating them with glutaminase inhibitors and assessing the labeling dynamics of [U–^13^C] glucose in downstream glycolytic metabolites.

Our proteomic analysis further revealed that cells 10 Days PTR upregulate key gluconeogenetic enzymes, FBP1 and PCK2 (Figure 1C). Santiappillai et al. reported this phenomenon in a subset of cancer cell lines that shuttled [U–^13^C] glutamine out of the TCA cycle into the gluconeogenic pathway, generating m+3 FBP and phosphoenolpyruvate (PEP) [74]. Others have reported cancer cells that perform glutamine-derived gluconeogenesis increase NADPH production for fatty acid synthesis and other biosynthetic precursors through PCK2 activity [75,76]. In our model, cells 10 Days PTR shuttle glucose- and glutamine-derived carbons to the oxPPP, generating 96% of their intracellular NADPH (Figure 5A–B). Importantly, our systematic quantification of major central carbon metabolism-related NADPH-generating enzymes revealed a metabolic switch in NADPH generation sources from malic enzyme-dependent production in parental cells (4.14 μmol/mg/hr) to PPP-dependent production in cells 10 Days PTR (17.50 μmol/mg/hr) (Figure 5A–B). This shift reflects a systems-level rewiring of NADPH generation rather than simply upregulation of antioxidant defense genes.

To our knowledge, this is the first study to apply isotope tracing and ^13^C-MFA to functionally characterize metabolism in cisplatin-surviving cancer cells that enter the non-proliferative polyploid cell state. While prior studies have reported changes in RNA expression and metabolite abundances in polyploid cancer cells in response to chemotherapy [6,10,12,73,77,78], our flux-based approach quantifies the magnitude, directionality, and kinetics of metabolic reprogramming – revealing not only which pathways are altered, but also how much flux is redirected and where nutrients are being utilized. This functional characterization provides mechanistic insights that cannot be inferred from gene expression or metabolite abundance data alone. Notably, while our ^13^C-MFA approach revealed the dramatic shift toward PPP-dependent NADPH production, stoichiometric calculations suggested that cells 10 Days PTR utilized 99% of this NADPH in metabolic pathways beyond central carbon metabolism, which our model could not resolve (Figure 5A–B).

To address this limitation, we applied genome scale metabolic modeling constrained by empirical metabolic data from cells 10 Days PTR and parental cells. To our knowledge, this is the first application of genome scale metabolic modeling to the non-proliferative polyploid cell state, and the first to integrate proteomics with ^13^C-MFA-derived flux constraints to predict systems-level metabolic compensation mechanisms in this context. Genome scale metabolic modeling is typically performed using flux balance analysis (FBA), which constrains extracellular fluxes and gene or protein expression data to maximize a user-defined cellular objective, such as ATP production or biomass growth [79]. However, the resulting fluxes are predicted by optimizing this objective function and may not accurately represent intracellular fluxes [79]. In this study, we integrated flux estimates from ^13^C-MFA based on data from isotope tracing of intracellular nutrient utilization into the genome scale model constraints to more accurately reflect the metabolic state of our cells. In addition, we performed flux sampling through CHRR-MCMC rather than optimizing for a singular cellular objective, allowing us to assess metabolic flux distributions at the genome scale in cells 10 Days PTR and parental cells with 95% confidence intervals [48].

Genome scale metabolic modeling revealed that cells 10 Days PTR increase antioxidant reaction fluxes when compared to parental cells (Figure 7B). These data align with our previous work and with Figure 6, showing that cells surviving cisplatin chemotherapy exhibit increased reactive oxygen species (ROS) levels over time and an enriched NRF2-mediated antioxidant response at the transcriptomic and proteomic levels when compared to parental cells [15,16]. Our previous work also demonstrated that the ratio of reduced glutathione (GSH) to oxidized glutathione (GSSG) increases over time as cells progress from 1 Day PTR to 10 Days PTR [16]. Additionally, cells 10 Days PTR exhibit elevated baseline lipid peroxidation as measured by C11-BODIPY staining when compared to parental cells, indicating sustained oxidative stress on lipid membranes [16]. The upregulation of the PPP in cells 10 Days PTR is particularly relevant in this context, as it serves as a major source of NADPH that is required by glutathione reductase to regenerate GSH from GSSG – a critical defense system against lipid peroxidation and ferroptosis [16,80]. Furthermore, we previously identified that inhibiting glutathione peroxidase 4 (GPX4) selectively induced ferroptosis in cisplatin-surviving cells, which has also been reported by other groups [16,81,82]. Despite their enhanced antioxidant capacity, the selective sensitivity of these cells to GPX4 inhibition is consistent with their elevated baseline lipid peroxidation and suggests that they are dependent on the PPP-NADPH-GSH-GPX4 axis for protection against ferroptosis. Future work involves testing the synergism of PPP and GPX4 inhibition to induce ferroptosis in cisplatin-surviving cells. Consistent with these findings, metabolic CRISPR screens have revealed that cancer cells surviving olaparib treatment via transient adaptive states are dependent on NRF2-driven antioxidant metabolism and the PPP, representing potential therapeutic targets [83].

When testing the essentiality of the oxPPP for antioxidant metabolism in cells 10 Days PTR in silico, we identified changes in other NADPH-producing and -consuming fluxes while detecting minimal loss of antioxidant fluxes. These simulations suggest that cells 10 Days PTR are metabolically plastic and can exhibit adaptive resistance to oxPPP inhibition. Notably, we observed elevated mitochondrial NADPH production via MTHFD in response to simulated oxPPP knockout, which could explain the increase in mitochondrial glutathione antioxidant activity through GTHRDt and GTHOm (Figure 7C and Supplementary Fig. 2A). Cells 10 Days PTR under oxPPP knockout also exhibited increased flux through malic enzyme (ME2) when compared to wild type cells 10 Days PTR (Supplementary Fig. 2A). Our findings are consistent with previous studies that reported cancer cells with a loss-of-function in G6PD increase flux through ME as a compensatory mechanism to generate NADPH [84,85].

Concurrently, we also observed decreases in NADPH-consuming fluxes related to folate metabolism – FOLR2 and MTHFR3 (Figure 7D–E and Supplementary Fig. 2B). We hypothesize that cells 10 Days PTR decrease folate metabolism as a compensatory mechanism to sustain intracellular NADPH pools for antioxidant activity. This is consistent with observations of folate deficiency and impaired dihydrofolate reductase (DHFR) activity in response to G6PD knockout across cancer cell lines [84]. As DHFR is the main enzyme that facilitates the FOLR2 reaction and generates tetrahydrofolate (THF), a precursor for MTHFD [46], we hypothesize that further inhibiting DHFR may induce depletion of THF that feeds into MTHFD, mitigating the elevated NADPH production we observed in cells 10 Days PTR under oxPPP knockout. Thus, residual DHFR activity may remain essential for survival in this resistant state.

These findings suggest a potential synthetic lethality treatment paradigm involving co-treatment of G6PD inhibitors, such as 6-aminonicotinamide or polydatin, and DHFR inhibitors, such as methotrexate, to eliminate cisplatin-surviving cells [[86], [87], [88]]. Testing this hypothesis with cell viability experiments will be important to assess synergism between G6PD and DHFR inhibition in cells 10 Days PTR. One concern of PPP inhibition is the potential on-target toxicities that would occur in vivo. Given that red blood cells rely heavily on the PPP for antioxidant metabolism, inhibiting G6PD could result in hemolytic anemia [66]. Encouragingly, the G6PD inhibitor, polydatin, demonstrated favorable tolerability in preclinical models (up to 200 mg/kg in rodents) and in a Phase II clinical trial (20–40 mg twice daily for 3 months) [[88], [89], [90], [91], [92]]. Another promising approach would be to target both G6PD and NRF2 to eliminate cisplatin-surviving cells. This strategy could be particularly relevant for patients whose tumors exhibit high PPP-Antioxidant expression (Figure 8A–D). Many NRF2 inhibitors are being developed in preclinical studies [93,94], and VVD-130037, a first-in-class Kelch-like ECH Associated Protein 1 (KEAP1) activator, is currently being tested in a Phase I clinical trial (NCT05954312) on patients with NRF2-driven tumors [95,96].

In summary, we propose that cisplatin-surviving PC3 cells exhibit a systems-level shift in central carbon metabolism to sustain antioxidant activity for cell survival. However, we acknowledge that this study has several limitations. First, we characterized metabolic reprogramming solely in cisplatin-treated PC3 cells, and the generalizability of these findings to other cancer types and treatments remains to be determined. Although cisplatin is not a first-line treatment for prostate cancer, PC3 cells are an androgen-independent model of metastatic castrate-resistant prostate cancer and exhibit certain neuroendocrine features – clinical contexts in which cisplatin is used [97,98]. Second, although we profiled the metabolic transcriptome at the single-cell level, bulk metabolomics may mask the metabolic heterogeneity within the surviving population. Profiling isotope tracer-based metabolomic differences at the single-cell level would be beneficial to capture potential coexistence of metabolic survival strategies in cells 10 Days PTR, but the available technology and methods are fairly new and need further development [99]. Third, we acknowledge that we did not perform perturbation experiments targeting these pathways in the current study. For example, direct quantification of intracellular ROS dynamics in response to PPP inhibition would provide direct evidence linking PPP-derived NADPH to ROS reduction. The contribution of NADPH to glutathione reductase activity was not experimentally tested, though the increasing ratio of GSH to GSSG over time we previously observed as cells progress from 1 Day PTR to 10 Days PTR is consistent with increased NADPH availability [16]. While direct perturbation of PPP enzymes would provide additional validation, our integrated approach – combining ^13^C-MFA, genome scale metabolic modeling, transcriptomic and proteomic profiling, ROS quantification, and clinical correlation – provides robust, multi-dimensional evidence for the functional importance of PPP-NADPH metabolism in cisplatin-surviving cells. This foundational metabolic characterization establishes the biological rationale and identifies specific vulnerabilities that inform therapeutic targeting strategies. Supporting the clinical relevance of these findings, our analysis of PPP-Antioxidant gene set expression from publicly available datasets revealed that the metabolic adaptations identified from cisplatin-surviving cells are clinically relevant and predict poor survival outcomes in patients across multiple cancer types (Figure 8A–D). While a previous study examined PPP gene signatures in cancer datasets [100], our integration of PPP and antioxidant pathway genes into a 20-gene signature represents a more comprehensive assessment of the metabolic phenotype that we observe. Our PPP-Antioxidant signature reflects the functional coupling between NADPH production (PPP enzymes) and consumption (antioxidant enzymes) revealed by our flux data, a connection that cannot be captured by examining PPP genes alone. This signature provides a clinically relevant biomarker panel grounded in functional metabolic data that captures both the source and utilization of NADPH in polyploid cisplatin-surviving cancer cells. This clinical association supports the significance of these metabolic pathways in disease progression. Fourth, while our data demonstrate that polyploidy and metabolic rewiring co-occur and are linked through NRF2-mediated transcriptional regulation, it would be valuable to explore the causal relationship between metabolic reprogramming and polyploidy to parse out whether these changes are directly due to polyploidy itself or represent parallel adaptations to cisplatin-mediated stress. Interestingly, the clinical association between our PPP-Antioxidant signature and poor outcomes in polyploidy-associated cancers suggests they are functionally linked. Determining the precise causal hierarchy represents an important direction for future mechanistic studies.

Despite these limitations, we believe our findings provide important groundwork for future research. We aim to expand these observations to other cancer model systems in which we observe the emergence of this non-proliferative polyploid cell state, such as MDA-MB-231 breast cancer cells, and with other chemotherapy treatments such as docetaxel and etoposide [3,5,7,16]. Comparisons with other non-genetic and adaptive models of therapy resistance, such as drug-tolerant persister cells, would provide additional context for understanding the heterogeneity of tumor cell evolution in response to treatment. Further characterization of the adaptive metabolic responses of the non-proliferative polyploid cell state across in vitro, in vivo, and patient-derived models with integrated fluxomics and functional validation will reveal new vulnerabilities that can be leveraged for targeting and improve patient outcomes.

## CRediT authorship contribution statement

**Melvin Li:** Writing – review & editing, Writing – original draft, Validation, Software, Methodology, Investigation, Formal analysis, Data curation, Conceptualization. **Bradley Priem:** Validation, Methodology, Investigation, Formal analysis. **Luke V. Loftus:** Validation, Investigation. **Michael J. Betenbaugh:** Supervision, Resources, Methodology, Funding acquisition. **Kenneth J. Pienta:** Writing – review & editing, Supervision, Resources, Project administration, Methodology, Funding acquisition, Conceptualization. **Sarah R. Amend:** Writing – review & editing, Supervision, Resources, Project administration, Methodology, Funding acquisition, Conceptualization.

## Funding

This work was supported by the 10.13039/100000005US Department of Defense 10.13039/100000090CDMRP/10.13039/100014039PCRP (W81XWH-20-10353 and W81XWH-22-1-0680), the 10.13039/100000892Prostate Cancer Foundation, and the 10.13039/100016737Patrick C. Walsh Prostate Cancer Research Fund to SRA; and NIH/NCI P01CA093900, and the 10.13039/100000892Prostate Cancer Foundation to KJP. This work was also supported by the Advanced Mammalian Biomanufacturing Center (AMBIC) through Industry-University Cooperative Research Center Program under 10.13039/100000001U.S. National Science Foundation grant number 1624684.

## Data availability

Data is provided within the manuscript or in supplementary information files. Source data for each figure is provided in Microsoft Excel format. Single-cell RNA sequencing data analysis was performed on a previously published dataset from our group that was deposited into the Gene Expression Omnibus (GEO) database (GSE297299). Patient RNA expression data used for Kaplan Meier survival analysis was downloaded from publicly available datasets from cBioPortal (https://www.cbioportal.org). The datasets used in this paper are listed as “Metastatic Prostate Adenocarcinoma (SU2C/PCF Dream Team, PNAS 2019)”, “Breast Invasive Carcinoma (TCGA, Firehose Legacy)”, and “Liver Hepatocellular Carcinoma (TCGA, Firehose Legacy)”. Proteomics data generated for this manuscript is deposited to ProteomeXchange via the PRIDE partner repository, with dataset identifier PXD073763. Code used to perform genome scale metabolic modeling is available at Github (https://github.com/mli154/genome-scale-modeling-with-proteomics-and-13C-MFA).

## Declaration of competing interest

The authors declare the following financial interests/personal relationships which may be considered as potential competing interests: Kenneth J. Pienta reports a relationship with PEEL Therapeutics that includes: equity or stocks. Kenneth J. Pienta reports a relationship with Krefect that includes: board membership and equity or stocks. If there are other authors, they declare that they have no known competing financial interests or personal relationships that could have appeared to influence the work reported in this paper.

## Data Availability

I have shared the link and information to research data and my code at the Attach Files step. Githubgenome-scale-modeling-with-proteomics-and-13C-MFA (Original data) cBioPortalMetastatic Prostate Adenocarcinoma (SU2C/PCF Dream Team, PNAS 2019) (Reference data) cBioPortalLiver Hepatocellular Carcinoma (TCGA, Firehose Legacy) (Reference data) cBioPortalBreast Invasive Carcinoma (TCGA, Firehose Legacy) (Reference data)
