# Supplementary figures and images for: Polyploid cancer cells surviving cisplatin reallocate central carbon sources to fuel antioxidant metabolism for survival
Source: Mol Metab. 2026 Apr 18;108:102370. doi: 10.1016/j.molmet.2026.102370 (PMC13158425; doi:10.1016/j.molmet.2026.102370)

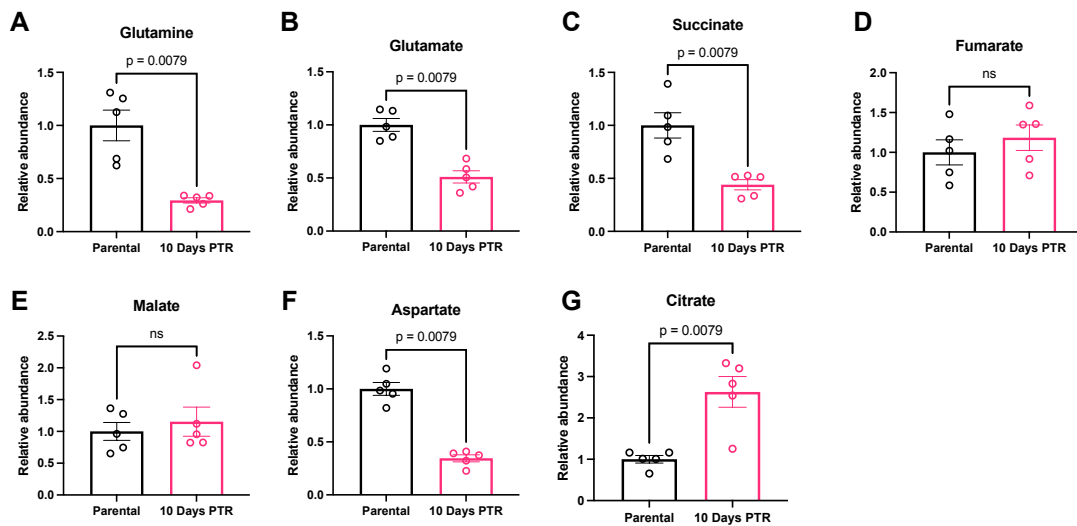

Supplement: Multimedia component 14 [file mmc14.pdf]

**A**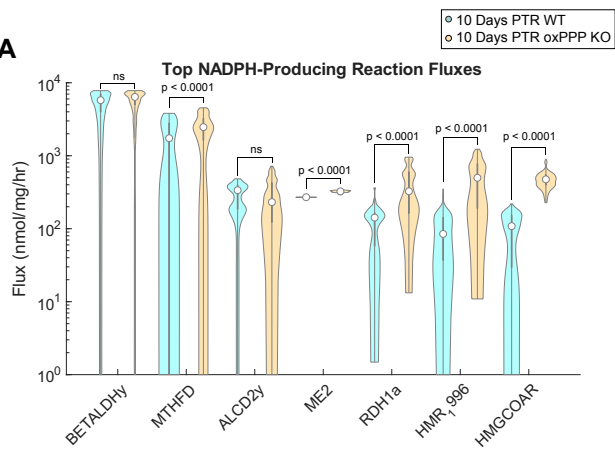**B**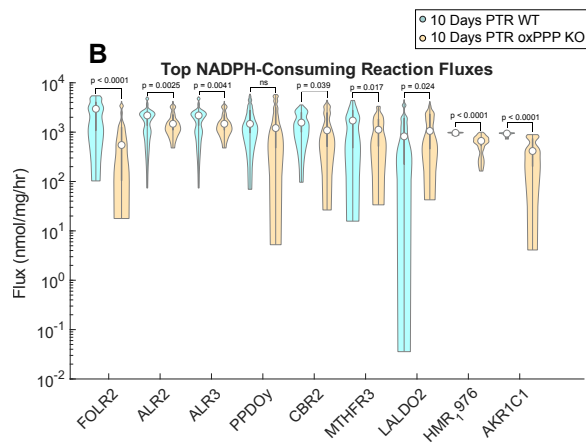

Supplement: Multimedia component 15 [file mmc15.pdf]
