# Supplementary material for: Polyploid cancer cells surviving cisplatin reallocate central carbon sources to fuel antioxidant metabolism for survival
Source: Mol Metab. 2026 Apr 18;108:102370. doi: 10.1016/j.molmet.2026.102370 (PMC13158425; doi:10.1016/j.molmet.2026.102370)
Supplement: Multimedia component 18 [file mmc18.docx]

**Description of Supplementary Files**

**Supplementary Figure 1. Relative pool sizes of TCA cycle metabolites in parental cells and Cells 10 Days PTR.** (**A**) Glutamine. (**B**) Glutamate. (**C**) Succinate. (**D**) Fumarate. (**E**) Malate. (**F**) Aspartate. (**G**) Citrate. n = 5 biological replicates for parental cells and cells 10 Days PTR. Data are presented as mean ± s.e.m. P-values were calculated using a two-tailed Mann-Whitney U test and exact values are indicated in the figure.

**Supplementary Figure 2. Cells 10 Days PTR exhibit increased NADPH production and decreased NADPH consumption upon *in silico* knockout of the oxidative pentose phosphate pathway.** (**A**) Sampled flux estimates of key NADPH-producing reactions between Cells 10 Days PTR wild-type (WT) and Cells 10 Days PTR oxPPP knockout (KO) (in units of nmol/mg/hr). (**B**) Sampled flux estimates of key NADPH-consuming reactions between Cells 10 Days PTR WT and Cells 10 Days PTR oxPPP KO (in units of nmol/mg/hr). n = 1000 CHRR samples per reaction, with 100 skips per CHRR sample. Data are presented as mean and interquartile range. P-values were calculated using boostrapped Mann-Whitney U tests. The median p-value for each reaction is indicated in the figure. Abbreviations: betaine-aldehyde dehydrogenase (BETALDHy), methylenetetrahydrofolate dehydrogenase (MTHFD), alcohol dehydrogenase (ALCD2y), malic enzyme (ME2), retinol dehydrogenase (RDH1a), 11Beta-hydroxysteroid dehydrogenase (HMR_1996), hydroxymethylglutaryl CoA reductase (HMGCOAR), folate reductase (FOLR2), aldose reductase (ALR2/3), propane-1,2-diol:NADP+ 1-oxidoreductase (LALDO2), 2-Oxo-5Alpha-steroid 4-dehydrogenase (HMR_1976), aldo-keto reductase family 1, member C1 (AKR1C1).

**File Name:** Supplementary Table 1

**Description**: Reduced Recon3D metabolic network reconstruction used for genome scale metabolic modeling. Number of reactions were reduced from 10,543 to 6,039 based on proteomic and ^13^C-metabolic flux analysis data. This table consists of reaction identifier in Recon3D nomenclature, the reaction name, and the reaction formula.

**File Name**: Supplementary Table 2

**Description**: Key antioxidant and NADPH-mediated reactions assessed from genome scale metabolic modeling between parental cells, Cells 10 Days PTR wild-type, and Cells 10 Days PTR oxPPP KO. This table consists of reaction identifier in Recon3D nomenclature, the reaction name, and the reaction formula.

**File Name**: Supplementary Data 1

**Description**: Input data of Cells 10 Days PTR wild-type fluxes for Escher map visualization at <https://escher.github.io/>.

**File Name**: Supplementary Data 2

**Description**: Input data of Cells 10 Days PTR oxPPP KO fluxes for Escher map visualization at <https://escher.github.io/>.

**File Name**: Supplementary Data 3

**Description**: Escher map .json file for visualization at <https://escher.github.io/>.

**File Name**: Source Data Figure 1

**Description**: Source data for Figure 1.

**File Name**: Source Data Figure 2

**Description**: Source data for Figure 2.

**File Name**: Source Data Figure 3

**Description**: Source data for Figure 3.

**File Name**: Source Data Figure 4

**Description**: Source data for Figure 4.

**File Name**: Source Data Figure 5

**Description**: Source data for Figure 5.

**File Name**: Source Data Figure 6

**Description**: Source data for Figure 6.

**File Name**: Source Data Figure 7

**Description**: Source data for Figure 7.

**File Name**: Source Data Figure 8

**Description**: Source data for Figure 8.

**File Name**: Source Data Supplementary Figure 1

**Description**: Source data for Supplementary Figure 1.

**File Name**: Source Data Supplementary Figure 2

**Description**: Source data for Supplementary Figure 2.
